# Systematic Review of Incidence of Cold-Welding Phenomenon in Use of Implants for Fracture Fixation and Collation of Removal Techniques

**DOI:** 10.3390/jcm14134564

**Published:** 2025-06-27

**Authors:** Fleur Shiers-Gelalis, Hannah Matthews, Paul Rodham, Vasileios P. Giannoudis, Peter V. Giannoudis

**Affiliations:** 1 Doncaster Royal Infirmary, Health Education Yorkshire and Humber, Doncaster DN2 5LT, UK; fleursg@icloud.com; 2 York District Hospital, York, Health Education Yorkshire and Humber, York YO31 8HE, UK; hannah.matthews9@nhs.net; 3 Academic Department of Trauma & Orthopedics, School of Medicine, University of Leeds, Leeds LS2 9JT, UK; plrodham@doctors.org.uk (P.R.); vasileios.giannoudis@doctors.org.uk (V.P.G.)

**Keywords:** cold-welding, metal implants, titanium implants, metalwork removal

## Abstract

**Introduction:** Cold welding is an anecdotally well-known complication of removal of metalwork, most commonly at the screw–plate interface, and can often complicate extraction of implants after fracture fixation. Even though this phenomenon is familiar amongst the orthopedic community, there is relatively little formalized discussion or literature pertaining to its identification and management clinically. In addition, as far as we can establish, there does not seem to be a paper that discusses the various techniques described in the literature that are employed to combat cold welding. **Methods:** A systematic review was carried out in accordance with the PRISMA guidance, with two independent reviewers and a third person to arbitrate for any discrepancies. Manuscripts were identified using a search of PubMed/MEDLINE and Google Scholar. Studies eligible for inclusion were tabulated and the results categorized qualitatively with respect to the technique described for removal of the implants. **Results:** A total of 272 manuscripts were identified using a search of PubMed/MEDLINE and Google Scholar, and of these 14 were ruled to be eligible for inclusion reporting on 292 patients. Common locations of the cold-welded screws included femur, tibia, distal radius and clavicle. The most common technique for metalwork removal was using either bolt cutters or burrs to cut the plates between the screws and mobilize the screw and plate as one unit. Other techniques included using specialized removal tools and cutting between the screw head and body. There was no appreciable correlation between the specific anatomic location of the welded implant and the technique used in its removal. From the studies, it was found that, of the total number of screws (n = 1654), 58 (3.5%) were cold welded. The mean time to metalwork removal was 1104 days (36.8 months). **Conclusions:** As far as we can tell, this is the first systematic review pertaining to the phenomenon of cold welding specifically, and with this project we have collated the techniques used to remove implants affected by cold welding from a variety of different articles. Our work aims to highlight the relative paucity of literature in this area and provide a number of accessible and safe techniques to facilitate the removal of cold-welded implants in fracture fixation.

## 1. Introduction

Cold welding, or ‘fusion’ as it is commonly referred to, is a phenomenon that can often cause problems for surgeons upon removal of metalwork or metal implants. Specifically, ‘cold welding’ refers to the unintended result of external pressure applied to two parts being joined, in the absence of heat, resulting in substantial plastic deformation [1].

On a molecular level, two clean surfaces are brought together to form intimate contact, generating strong bonds at the interface [2]. This occurs as a result of disruption of the surface oxide layers and intimate molecular contact between metal atoms, leading to metallic adhesion [1]. Cold welding is thought to occur due to incorrect or excessive torque applied to the screw head, errors in manufacturing of the implant/screwdriver, or the presence of bone debris or dried blood on implant surfaces leading to the development of a fibrin ‘glue’ [3].

The clinical significance of this arises in procedures involving the removal of metalwork. This is a commonly performed procedure in both the elective and trauma setting. This information is not readily available with data from the UK. However, a Finnish study showed that removal of metalwork accounted for 30% of the elective operating case load in Finland [4], and it has been estimated to comprise 5% of all orthopedic surgery procedures performed annually in the USA [5]. In the context of arthroplasty, cold welding has been described specifically regarding modular component junctions in total hip [6,7,8,9,10] and shoulder replacements [11], which has complicated revision surgeries. In our review, we also found cases of cold welding between trapeziometacarpal ball and socket prostheses [12]; however, these cases are significantly different to cold welding in the context of trauma and fracture fixation and were therefore excluded from our analysis.

It is commonplace for removal of metalwork procedures to be carried out by more junior orthopedic surgeons and is often a procedure underestimated in its complexity. Removal of metalwork can be difficult to carry out due to equipment issues and breakage of screws, and implant removal can take longer to perform than the index procedure, resulting in complications [13]. Cold welding can significantly contribute to the increased complexity of such cases, and there is relative paucity of literature regarding how to manage this and, until now, no study has aimed to collate the instances of cold welding identified during implant removal, and, more importantly, how the surgical teams managed to remove the metalwork.

Currently, there is no clear-cut guidance on when metalwork removal should be undertaken in adults [14]. In contrast, in children, removal of metalwork is more readily performed, especially when metalwork is near the physes or if there are restrictions in range of movement that may be attributed to the metalwork. In the adult population, it is generally accepted that metalwork can and should be removed in the context of complications such as non/mal-union, implant breakage, infections, conversion to arthroplasty procedures and local symptoms [15], but it remains a contentious subject. There is even less guidance regarding the management of intraoperative complications during implant removal, and specifically that of cold welding.

The aim of this study therefore was to collate the evidence in the literature investigating current practices of managing removal of hardware associated with the phenomenon of cold welding.

## 2. Methods

### 2.1. Literature Search Strategy

A comprehensive search was undertaken according to the Preferred Reporting Systems for Systematic Reviews and Meta-Analysis (PRISMA) statement [16] using the search terms “(cold-welding OR cold welding, OR cold fusion AND metal implants) OR (titanium implants OR metalwork removal)”. The search was carried out using PubMed/MEDLINE and Google Scholar and identified papers between 1 January 1989 and 22 October 2024. The search strategy is outlined in Appendix A.

The articles identified by the initial search were screened for relevance according to our eligibility criteria by two independent reviewers (FSG, HM), and then further exclusions were made after reading the main body of the texts. Where there was disagreement about whether to include an article, a third independent reviewer (VPG) acted as an arbitrator and made the final decision.

There was no blinding of reviewers to authors, journals, or institutions.

### 2.2. Eligibility Criteria

Studies were included in our analysis if they met the following criteria.

1. Case reports, systematic reviews, retrospective case series and prospective cohort studies specific to the phenomenon of cold welding or cold fusion of metal or titanium implants in humans.
2. Reported cold welding or cold fusion in the context of the use of implants for fracture fixation.
3. Any paper describing methods of removal of cold-welded or fused implants in adults and children.

Studies were excluded from our analysis if any of the following criteria were met:

1. Animal or non-human studies.
2. Dental/oral surgery studies.
3. Any other metalwork removal issue such as cross-threading, screw head stripping or equipment failure.
4. Articles not written in the English language.

### 2.3. Assimilation of Data

Each relevant paper was analyzed and specific data points extracted; sample size, type of implant, number of screws affected, anatomical location and techniques described for removal. Where cold welding was mentioned in association with other metalwork removal issues such as cross-threading, care was taken to only analyze numbers of screws/implants deemed by the authors to be cold welded.

### 2.4. Statistical Analysis

Data extracted was inserted into SPSS (IBM Corp. Released 2017. IBMSPSS Statistics for Windows, Version 25.0. IBM Corp.: Armonk, NY, USA) and analyzed. The heterogeneous nature of the data collected allowed us only to perform descriptive statistics. Statistical significance was assumed at a *p*-value of less than 0.05.

## 3. Results

Our search yielded 272 articles initially. After we removed duplicates and then screened the titles and abstracts, 25 appeared to meet the inclusion criteria for further analysis. Four papers were subsequently excluded due to a lack of a description of the removal technique specifically utilized for cold-welded implants. After discussion with a third reviewer, the decision was made to exclude 7 papers that dealt with the concept of cold fusion in the context of arthroplasty surgery, leaving a total of 14 [13,17,18,19,20,21,22,23,24,25,26,27,28,29] papers for in-depth analysis. A further paper was put forward by a third reviewer pertaining to an incidence of cold welding of a proximal lag screw discovered upon removal of a proximal femoral nail [29].

**Figure 1:**
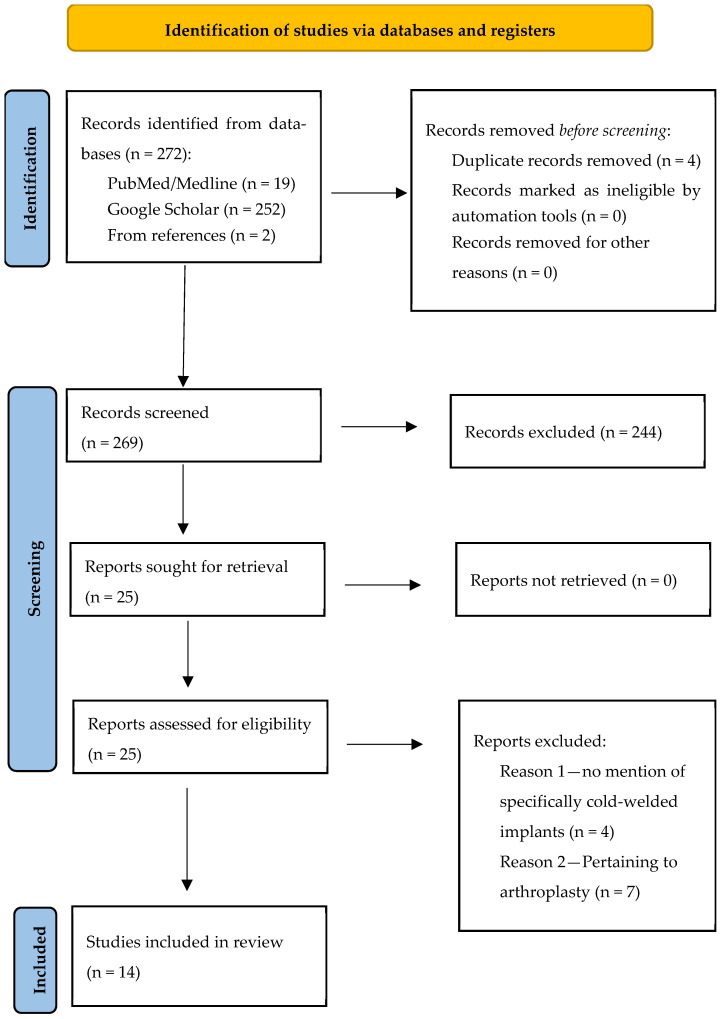
PRISMA flowchart of cold-welding studies.

The PRISMA flowchart summarizes the search strategy used (Figure 1).

Five of the papers excluded reported incidences of cold welding in hip arthroplasty between the articulating head and the trunnion [6,7,8,9,10]. One paper discussed cold welding in the context of trapeziometacarpal ball and socket prostheses [12] and another pertaining to reverse total shoulder arthroplasty [11].

Therefore, a total of 14 papers were included in the study reporting on 292 patients. All papers described episodes of cold welding that had occurred secondary to fracture fixation and are shown in Table 1 [13,17,18,19,20,21,22,23,24,25,26,27,28,29]. They discussed cold welding in terms of screws becoming cold welded to plates, except for one case report describing the difficulty of removing a cold-welded proximal lag screw from a proximal femoral nailing system [29]. Noteworthy, 12 out of the 14 papers discussed a technique for overcoming cold welding [13,17,18,19,20,21,22,23,24,25,26,27]. However, two of them gave no case examples of how the authors had implemented the technique, only that they would endorse removal of the metalwork with the technique discussed. The remaining paper discussed the event of cold welding but did not discuss how the authors managed this complication [28].

The most common type of publication describing cold welding or cold fusion were isolated case reports totaling seven papers [17,18,21,22,24,25,26].

The techniques described for the removal of cold-welded implants included using various burrs and saws to cut either around the screw to free it from the plate or cutting the plate into sections to remove the screw and plate ‘on bloc’, and the use of specialized removal tools. There was no appreciable correlation between the specific anatomic location of the welded implant and the technique used in its removal. Furthermore, the mean time to metalwork removal was 1104 days (36.8 months).

Common locations of the cold-welded screws included femur, tibia, distal radius and clavicle. We are unable to provide accurate statistical analysis with respect to the incidence of cold welding at the plate/nail screw interface as the authors in all the studies did not provide details of how many screws were used with each fixation device. From the studies that provided the total number of screws (n = 1654) and number that were cold welded, the incidence was 58 (3.5%).

Titanium is by far the most commonly reported material to demonstrate cold welding, with 9/14 articles specifying the material of the plates as titanium [17,18,19,20,21,23,26,28,29]. Four articles [13,22,25,27] did not state the material explicitly, and one reported cold welding of a sliding hip screw to the barrel of a stainless-steel implant [24].

Eleven articles [13,17,18,19,20,21,22,24,25,28] specified reasons for metalwork removal. The most common reason stated for removal of metalwork was painful/symptomatic implants, accounting for 154/292 (52.7%) cases of metalwork removal. Furthermore, non-union cases accounted for 21/292 (7.2%), with patient preference accounting only for 6/292 (2.1%) cases of metalwork removal. Of note, three of the papers we found did not include this information as part of their narrative.

The majority of articles we analyzed did not reference complications associated with the removal of cold-welded implants. However, Raja et al. [13] and Suzuki et al. [20] reported complication rates of 47% and 38.9%, respectively, without specifying whether or not these complications were present in cases with cold-welded implants. In Suzuki et al. [20], superficial infection and transient peroneal nerve palsy were listed as complications post-surgery. Both Lehmen et al. [21] and Nortwick et al. [18] reported significant metal debris in conjunction with attempted removal of a cold-welded implant. All but one paper reported complete removal of metalwork; however, Singh et al. [22] were able to remove the heads of the screws but left the threaded portion in situ. Table 1 includes the results of the literature that discusses cold welding cases.

The percentages reported in our results table (Table 1), are based on denominators available for each individual study. Please note some denominators are not listed in view of the fact this information was not provided in the source material. Where specified, the denominator refers to the number of total screws used in the cohort, and pooled incidence calculations were only carried out where data from studies explicitly reporting both total screws used and the number of those screws that were cold welded was available. No weighting or statistical analysis could be applied due to the heterogeneity of the data (Table 2).

## 4. Discussion

This study illustrates a number of interesting factors regarding the incidence and management of cold welding amongst metal implants. Looking at the number of papers identified that explored the cold welding of implants and the effect on metalwork removal, there were a total of 24 studies, which is an objectively small number when considering how common place metalwork removal is, and how frequently cold welding is thought to take place. Of these, even fewer studies met the eligibility criteria for inclusion in this review, and an even smaller number discussed cold welding separately to other metalwork complications. It is also interesting to note the frequency of studies that appear to conflate the issues of screw head ‘stripping’ with cold welding, as these are different complications that need different management. A screw head that is merely stripped in theory may be extracted with an Operace screw extraction set, but a screw that is cold welded to the plate on a molecular level has become bonded by definition, and therefore it is our view that attempting to remove the screw head from the plate is possibly a wasted endeavor. This is reflected in our research as the most successful methods of cold-welded implant removal involved the cutting of the plate and removal of the affected screw still attached to that portion of the plate.

The paucity of literature pertaining to cold welding in and of itself is an interesting finding, especially given the prevalence of metalwork removal. This may be due to the idea that metalwork removal and its resultant common intraoperative complications such as cross-threading of screw heads and screw breakage are seen as insignificant events in orthopedic surgery, despite the evident difficulties that arise from them. Removal of metalwork can carry significant risk to the patient, not only intraoperatively but also in the postoperative period, and complications such as infection, nerve damage, scarring, further fracture and retained metalwork are well documented. As a result, removal of metalwork should not be undertaken without obvious clinical need and should be done with as much preparation as possible.

As mentioned in the results, seven of the papers [6,7,8,9,10,11,12] discussed specifically cold welding in the context of arthroplasty and were therefore not included in our analysis; however, it is interesting to note that a significant proportion of the literature pertaining to the cold-welding phenomenon describes cold fusion between the articulating head and the trunnion, which may have interesting insights for the purposes of revision surgery.

The most common technique for managing an implant that was cold welded was some iteration of cutting the plate between the screws and attempting to remove the screw and plate section as one using high-speed cutters such as the Midas Rex cutter or carbide burrs. No failures were reported using this technique, and it seems to be something that could be easily reproducible in most theatres as there is no need for specialized equipment. Another two papers [21,22] detail attempts to combat cold welding specifically focusing on the screws, either by cutting off the screw head to free it from its body or using an instrument such as Jacob’s T-handle chuck connected to a Synthes star driver shaft, and then placing a screwdriver into the screw head, incarcerating and then turning the handle. These techniques may involve slightly more technical difficulty and require the use of adjuncts in the removal of the screws and risk damaging them in the process. In addition, discussion surrounding metal debris was commonplace when describing various removal techniques, and an important consideration when using equipment such as a specialized burr is the generation of copious amounts of metal debris, highlighting the importance of protecting the surrounding tissues while using the burr or saw.

Another interesting facet to the discussion surrounding cold welding and metal implants is the discrepancy in the literature identified regarding whether or not it is a more commonplace occurrence in titanium or stainless-steel implants. In our review, we identified that one author stated that cold welding was more ‘problematic’ in titanium implants [20], and another reported that in their experience cold welding was more likely to occur between two ‘perfectly polished’ surfaces like those between stainless steel implants, and was unlikely to occur in titanium [30]. Work has been done to develop titanium alloys for orthopedic implants [31] in order to ‘eliminate’ the risk of cold welding by reducing adhesion at a molecular level [32].

In addition, our wider search of the literature did not demonstrate any instances of cold welding in carbon fiber implants. However, one paper [33] detailed adverse events in the use of CFR-PEEK plating for the fixation of distal radius fractures, which most commonly consisted of plate rupture but no incidences of cold welding.

It is clear from our research that the most commonly performed technique for the removal of a cold-welded screw is to cut the plate either side of the screw and rotate anticlockwise in order to remove the screw/plate as one unit. An important consideration is the mechanism by which the plate is cut—it is preferable to use a technique that produces as little metal debris as possible, preferably using metal cutters. If this is not achievable, and a carbine burr needs to be used, steps should be taken to minimize soft tissue contamination with metal debris by using techniques such as the utilization of a saline-soaked gauze or careful irrigation.

**Figure 2:**
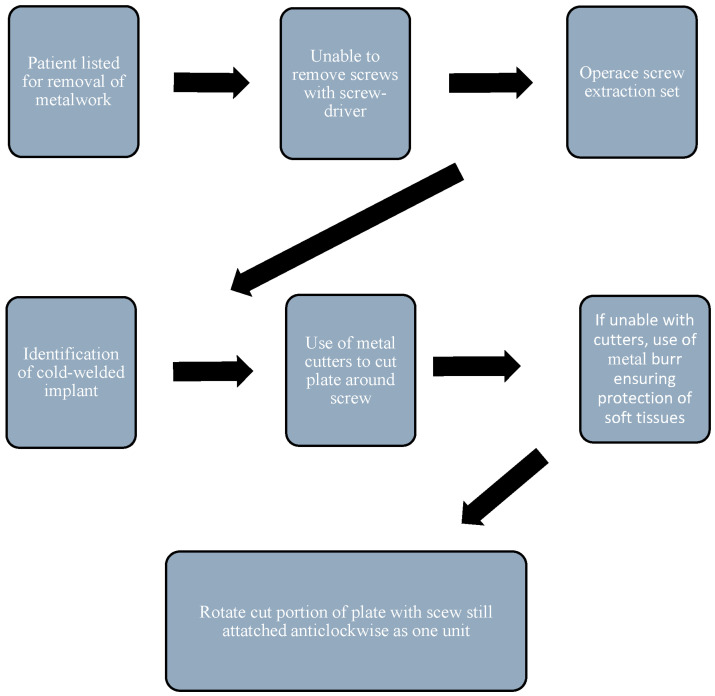
Flow chart showing the steps to identify and remove cold welded implants.

We have derived a step-by-step guide that summarizes what we feel are the best management options for the removal of a cold-welded implant (Figure 2). An important consideration for the surgeon is to ascertain whether they believe the difficulty in removing the implant is attributable to screw stripping, cross-threading or cold welding, as this will determine the best way to manage extraction.

## 5. Limitations

Our study has some limitations, primarily the paucity of literature published, and there is a lack of research into cold welding or cold fusion specifically, as opposed to general complications that arise from metalwork removal. Moreover, the studies we did find are relatively underpowered, with case reports making up the bulk of contributions to the data, which is anecdotal evidence.

Another limitation to this review is the lack of chemical analysis in the identified articles to confirm the presence of cold welding. Cold welding was attributed to the implant by the authors but there is no way to chemically confirm its presence as opposed to the screw/implant being cross-threaded or jammed in place. In addition, one study [20] identified used the terms ‘screw stripping’ and ‘cold fusion’ interchangeably, which may produce a bias as ‘screw stripping’ is felt to be an altogether different complication.

## 6. Risk of Bias

For our review, it was challenging to use a formal risk of bias tool due to the study types identified in this study. The papers we discuss in this review are primarily case reports and retrospective case series, which our preferred Critical Appraisal Skills Program Checklist (CASP) does not recommend for this study type.

The majority of reports included are of a retrospective and descriptive nature, without randomization, blinding or control grouping. Most cases discussed in the literature we found were published due to their complexity of implant removal, which might result in an overrepresentation of the more severe/complex instances of cold welding, resulting in some selection bias.

Performance bias cannot be assessed due to a variance in techniques for implant removal, along with inconsistent description of the techniques used, and no universal standardized protocol being utilized.

As we discuss in our review, the lack of chemical analysis of cold welding and objective confirmation of its presence results in a certain degree of detection bias, as the diagnosis of cold welding in the studies we include were all made clinically.

In addition, we note that a number of papers we analyzed provided incomplete information regarding patient characteristics, implant specifics and longer-term outcomes of these removal techniques, increasing the risk of reporting bias. The reporting of complications was limited to a subset of papers included in this review, which may have resulted in their underrepresentation in general, which is another factor to consider.

In summary, our review is at risk of a moderate degree of bias, due to the nature of the reports and papers analyzed, specifically their anecdotal and retrospective characteristics, along with the variability in reporting we encountered. This serves to further underline the need for more robust and standardized research in this area. However, we acknowledge that a limitation of this review is our inability to formally quantify this bias risk.

## 7. Conclusions

Cold welding is a relatively common complication discovered intraoperatively during removal of metalwork operations and can be difficult to manage. Depending on the type of implant affected, the anatomical location of the implant and equipment available to the team, several options exist for how to manage this problem. The most common method of extraction of a cold-welded implant is the use of a burr to split the plate between the screws to make screw/plate ‘units’ and remove the units individually. Cold welding is an important factor to consider when undertaking a removal of metalwork list, and it is important to have knowledge of this phenomenon and have strategies to manage it that are accessible and safe.

## Figures and Tables

**Table 1:** Results of literature discussing cold welding cases.

Paper	Study Design	Number of Subjects in Study	Date of Metalwork Removal (^a^)	Reason for Metalwork Removal	Number of Incidences of ‘Cold Welding’ (%)	Anatomical Location of Cold-Welded Screws	Type of Plate/Screw	Material of plate/screw	Removal Technique	Complications
Raja et al. (2012) [13]	Retrospective case series	37	5–588 days (mean 507.8 days)	Painful/palpable implant (n = 17), non-union (n = 9), implant failure (n = 3), avascular necrosis (n = 3), patient demand (n = 2), facilitate other procedure (n = 2), infection (n = 1)	10 (no total screw number given), 8 cases affected (22.2% of cases)	Proximal tibia (5) Distal tibia (1) Femur (1) Humerus (1)	Locking plates Proximal tibial LISS plate Distal femoral LISS plate Distal tibial LCP plate PHILOS plate	Not stated	Pneumatic high-speed metal cutting burr to cut around screw	Complication rate 47% (no detail given) All cold-welded screws removed—one still attached to plate
Gopinathan (2013) [17]	Case report	1	1 year	Skin tenting	5/6 screws (83.3%)	Clavicle	Locking recon plate	Titanium	Bolt cutter to divide plate into sections, screws removed with plate attached	
Nortwick et al. (2012) [18]	Case series	2	5 years	Painful implant	3/10 screws, 1 case affected (30%)	Distal radius screw removal from plates	DVR Volar locking plate	Titanium	High-speed cutter (Midas Rex) used to divide plate into sections, screws removed with plate attached	Incomplete removal of titanium debris from soft tissues
Bakane et al. (2023) [19]	Retrospective case series	30	Not stated	Further trauma or disease (41.7), painful implant (28.6) and patient preference (11.9)	Not stated	Femoral, humerus, clavicle and tibia	LCP—locking plates	Titanium	Carbide drill to drill out the head then extract the shaft Cut the plate around the screw, releasing it. Cut the plate into sections and remove the plate screw in combination anticlockwise	
Suzuki et al. (2010) [20]	Retrospective case series	33	Average 13.2 months	Symptomatic implants (n = 21), non-union (n = 12), ‘loss of fixation’ (n = 2), peri-implant fracture (n = 1)	37/349 (10.6%) referred to as ‘difficult removals’ requiring cutting of the plate. “Screw stripping” attributed to cold welding in this paper	Femur (n = 21) Tibia (n = 15)	LISS locking plates	Titanium	Carbide/diamond tipped burr, bolt-cutter, to divide plate into sections, screws removed with plate attached. Conical reverse-threaded extraction device to remove screw	Postoperative infection (n = 2) Peroneal nerve palsy (n = 1) Complication rate reported as 38.9%
Lehmen et al. (2011) [21]	Case report	1	Not stated	Painful implant	6 screws cold welded—total number of screws not stated	Distal tibial	LISS tibial plate—locking screws	Titanium	First 3 cold welded screw heads removed with carbide burr. Subsequent 3 screws removed with Jacob’s T-handle chuck connected to Synthes star driver shaft, screwdriver placed into screw head, and incarcerated, then turned	Significant metal debris with first technique
Singh et al. (2015) [22]	Case report	1	3 years	Non-healing ulcer	4/5 ‘jammed’ screws (80%). Not specifically termed as ‘cold-welded’	Distal tibia	Distal tibial locking plate	Not stated	Stainless steel metal cutting blades used to cut the screw between the plate and the bone leaving threaded part of screw in situ	Threaded parts of 4 screws left in bone, unable to remove after removal of plate
Garg et al. (2011) [23]	Retrospective case series	27	Mean time 2.3 years	Not listed	None mentioned as ‘cold-welded’ but referred to as ‘jammed’ 15/248 screws (6.04%)	Not specified correctly—numbers do not add up or specify whether cold welded or not	Range of locking plates	Titanium	Cutting plate into sections using saw initially to weaken it, then remaining portion with metal cutter (to avoid bone damage), screw removed with plate attached	Postoperative infection (n = 2), postoperative fracture (n = 1)
Sreenivasan et al. (2016) [24]	Case Report	1	10 years	Peri-implant fracture	1	Proximal femur	Sliding hip screw	Stainless steel	Rotation of plate-screw construct as a whole like a spanner	
Agrawal et al. (2018) [25]	Case report	1	3 years	Painful implant	1/6 (16.7%)	Clavicle	Clavicle plate	Not stated	Plate cut and bent then rotated anticlockwise	
Dimock et al. (2019) [26]	Case report	Not stated	Not stated	Not stated	Not stated	Distal femoral screw removal from plate	Locking plate but not specified	Titanium	Presentation of a new technique—a high-speed carbide burr used to divide plate into sections, screws removed with plate attached then twisting them anticlockwise they coined the ‘helicopter technique’	
Kumar et al. (2020) [27]	Expert opinion	Not stated	Not stated	Not stated	Not stated	Not stated	Locking plates but not specified	Not stated	Retrograde—locates the tip of the screw and uses a novel device—T handle with a shaft 2 mm larger than the screw threads to rotate it out of the plate	This is a presentation of a novel device to combat cold welding, no real-life examples given
Dehghan et al. (2024) [28]	Retrospective case series	157	Mean time 467 days	Irritation (66%), infection (20%), failure/revision (10%)	8/1274 (0.6%)	Upper and lower extremities included	Variety of locking and non-locking plates	Titanium	No technique just observation of cold welding	
Yanagisawa et al. (2021) [29]	Case report	1	18 months	Loosening of femoral lag screw	1	Proximal femur	Trigen Meta-Tan nail	Titanium	Carbide drill to cut lag screw and outer surface of nail	

^(a)^ How long after initial procedure metalwork was removed.

**Table 2:** Summary of techniques described in the literature.

Paper	Technique Described
Kumar et al. [27]	Novel device—T handle with a shaft 2 mm larger than the screw threads to rotate it out of the plate
Dimock et al. [26], Suzuki et al. [20]	High-speed carbide drill used to divide plate into sections. Screw/plate mobilized as single unit by turning anticlockwise
Agrawal et al. [25], Sreenivasan et al. [24], Gopinathan [17]	Plate cut, bent and rotated anticlockwise as one plate/screw construct
Garg et al. [23]	Plate cut into sections initially with saw, remaining portion cut with metal cutter. Screw/plate construct mobilized as one
Singh et al. [22]	Stainless steel metal cutting blades cut screw between the plate and the bone leaving threaded part of screw in situ
Lehmen et al. [21]	Jacob’s T-handle chuck connected to Synthes star driver shaft, screwdriver placed into screw head, incarcerated, then turned
Bakane et al. [19]	Carbide drill, drill out head, extract shaft. Cut the plate around the screw to release
Nortwick et al. [18]	High-speed cutter (Midas Rex) divide plate into sections, screws removed with plate attached
Raja et al. [13]	Pneumatic high-speed metal cutting burr to cut around screw
Yanagisawa et al. [29]	Carbide drill to cut lag screw and outer surface of nail

## Data Availability

No new data was generated for this article. Existing studies were analyzed as part of this review, and their details can be found in the list of references.

## Appendix A

**Table A1:** Search Strategy.

MEDLINE 22 October 2024	Google Scholar 22 October: 2024
1. cold-welding/	1. “cold-welding”/
2. cold welding/	2. “cold welding”/
3. cold fusion/	3. “cold fusion”
4. 1 or 2 or 3	4. 1 or 2 or3
5. metalwork removal/	5. “metalwork removal”/
6. metal implant	6. “metal implant”/
7. titanium implant	7. “titanium implant”
8. 5 or 6 or 7	8. 5 or 6 or 7
9. 4 and 8	9. 4 and 8
